# Incidence Trends of Cancer in Morocco: The Tale of the Oncological Center of Marrakech (Morocco) over 8 Years

**DOI:** 10.1155/2022/3307194

**Published:** 2022-02-28

**Authors:** Rhizlane Belbaraka, Nada Benhima, Ahmed Laatabi, Mohammed El Fadli, Ismail Essâdi

**Affiliations:** ^1^Medical Oncology Department, Mohammed VI University Hospital, Marrakech, Morocco; ^2^Faculty of Medicine and Pharmacy, Cadi Ayyad University, Marrakech, Morocco; ^3^Mathematics and Population Dynamics Laboratory, Cadi Ayyad University, Marrakesh, Morocco; ^4^Medical Oncology Department, Avicenne Military Hospital, Marrakech, Morocco

## Abstract

**Background:**

Determining cancer incidence and mortality is a key factor in the implementation of health policies and cancer prevention strategies. This report aims to describe the trends of cancer incidence in a single referral oncology department from the Marrakech region (Morocco). *Material and Methods*. All new cancer cases of age ≥ 15 years registered at the Medical Oncology department of Mohammed VI University Hospital of Marrakesh between January 1, 2012, and December 31, 2019, were included. Central nervous system (CNS) cancers, tumors of hematopoietic and lymphoid tissues, and thyroid cancers for which chemotherapy was not indicated or was managed in other cancer-specialized departments were excluded from the analysis. Manual data collection from printed archived medical records of the study population was performed. Descriptive statistics were analyzed using R software and Joinpoint Regression Program.

**Results:**

A total of 15648 new cancer cases were analyzed. Missing data (*n* = 1822) accounted for 11.64%, and 4.1% (*n* = 652) were excluded. The final statistical analysis and registration included 13174 cases. The median age at diagnosis is 54 years for females and 61 years for males. Female patients outnumbered males with a ratio of 1.58 among all age groups except those aged ≥75 y. The age-standardized incidence rate (ASIR) for all sites was 68,0 per 100.000 person-years, which has increased with an annual percent change (APC) of 10.61%. The five most common malignancies among males are lung, stomach, prostate, colic, and rectal cancers. Among females, the five most frequent cancers are breast, cervix, ovary, colon, and stomach.

**Conclusion:**

The higher incidence observed in our results translates into a growing burden on the center and is expected to impact our ability to deliver cancer care. Epidemiological studies to identify risk factors and effective efforts are needed to further invest in cancer control and prevention plans.

## 1. Introduction

Global statistics reveal increasing threats about the burden of cancer in African countries. Surprisingly, cancer data sources in the continent are scarce. The percentage of the African population covered by cancer registries of sufficient quality to appear in the series of Cancer Incidence in Five Continents (CI5), regarded as a longstanding and ultimate source for high-quality cancer incidence data, has not exceeded 2% [1]. The most recent volume (volume XI) includes datasets from only seven population-based cancer registries (PBCRs), based in six African countries (Algeria, Kenya, Seychelles, South Africa, Uganda, and Zimbabwe) [2].

Despite several facilities provided by regulatory agencies to pursue sustainable and efficient data collection programs, the obstacles that thwart the establishment of cancer registries in Africa are still elusive. The challenge is how to adapt these settings to the reality of limited resources, insufficient funding, the continuous use of manual methods, and the limited qualified staff all over the continent.

In Morocco, providing efficient resources for cancer registration has been at the core of the national cancer plan for cancer control. Heretofore, the national incidence is derived from the two available population-based registries of the Casablanca and Rabat regions. The first report from Casablanca, covering around 12% of the Moroccan population, provides incidence of all types of cancer collected retrospectively from hospital departments, private hospitals, and pathology laboratories in the region [3]. The second registry from Rabat, covering around 2.1% of the population, used an active data collection process involving visits by registry staff to public and private facilities where new cancer cases were diagnosed or treated [4]. The most recent reports have covered the period from 2009 to 2012. Thus, the national incidence is based on a local weighted average and applied to the global population [5]. However, given the modest coverage over the country, the accuracy of these registries in reflecting the national incidence of cancer is insufficient and should be interpreted with a degree of caution.

The scarcity of published data from local hospitals complicates the situation further and requires a special review effort to understand the epidemiology of cancer in Morocco. Hospital-based cancer registries (HBCRs) provide annual reports on the burden of cancer in all patients referred to a single hospital. Ensuring high-quality data collection, analysis, and reporting from hospital registries may provide necessary and effective resources for population-based cancer registries and sustainable cancer-related information for clinical research.

To our knowledge, there has been no previous published data on cancer incidence in the Marrakech region. In this study, we report the cancer incidence and the frequency distribution of cancer cases according to the site, year of diagnosis, sex, and age groups of patients admitted to a single Medical Oncology Department in the public tertiary hospital of Marrakech.

## 2. Material and Methods

### 2.1. Sources of Data

Mohammed VI University Hospital is the largest of the four hospitals in Marrakech and the only pubic facility with a referral cancer unit in the region. Situated in central Morocco, the Marrakech region covers an area of 31,160 km^2^ and has a population of 4,504,767 (2014 census). Marrakech city, having an extended population of 1.3 million, is the second most populated city in the country. The hospital admits all referred cancer cases from other healthcare facilities in the region and its surrounding areas, mainly from the southern regions.

This registry is based on the records of the single Medical Oncology Department. In the absence of a shared registry of all cancer cases referred to the hospital, we had no access to the records of the patients with pediatric cancers, tumors of hematopoietic and lymphoid tissues, and most thyroid cancers that are treated, respectively, in pediatric oncology, hematology, and nuclear medicine-specialized departments. We also had no access to the records of the patients with brain cancers admitted at the department of radiation oncology who were elected for exclusive radiotherapy without indication of concomitant chemotherapy.

All new cancer cases aged ≥15 years admitted at the Medical Oncology Department of the university-affiliated hospital were included. The inclusion period covered eight years from January 1, 2012, to December 31, 2019. The date of incidence was primarily defined as the date of the first admission in the Medical Oncology Department, posterior to the date of histological diagnosis. Malignant neoplasms were defined based on the International Classification Diseases for Oncology (ICD-O) in its 3rd edition-2nd revision [6].

Manual data collection from paper-based inpatient records was performed by medical residents trained on cancer registration. The patient identifier was used as a reference for checking duplicated cases. The collected data included patient registration number, age, sex, date of diagnosis, and primary site of cancer. New cancer cases were counted for each primary tumor. Patients diagnosed with multiple primary malignancies, defined as synchronous or metachronous primary malignant tumors of different histological origins, appear more than once in the dataset. Secondary tumors and recurrences of a previous cancer were not counted as new cases. Cancers *in situ* which progressed to be invasive at a later stage were registered in the year they were diagnosed as invasive carcinomas. Treatment information and follow-up data were not collected.

### 2.2. Statistical Analysis

Data were entered, processed, and analyzed using R software and Joinpoint Regression Program (version 4.8). The results are presented as the number of cases by cancer site (ICD-O-3.2), sex, year, and age. Categorical data are provided as frequencies and percentages and continuous variables as means with standard deviations. Based on the official data of the High Commission for Planning (HCP) [7], the adult population (≥15 years) in the region was 3,143,792 people in 2014, including 1,573,866 males (50.1%) and 1,570,338 females (49.9%). The age groups for each type of cancer are constructed as follows: 15-29 y, 30-44, 45-59, 60-74, and 75+.

Crude incidence rates (CIR) represent the number of new cancer cases over the 2014 population (we consider 2014 as the mid-year population of 2012-2019 as it is the year where an official census is available). Age-standardized incidence rates (ASIR) represent the rates that would be observed if our population had the same age structure as the standard reference population (Segi-Doll world population [8, 9]). ASIRs are used to compare our findings with other published registries reporting data from different countries and periods. The standard error measures the statistical accuracy of ASIRs. CIRs and ASIRs are calculated per 100,000 person-years.

The annual percent change (APC) is used to characterize incidence trends in ASIRs over the 8-year study period. The JoinPoint Regression Program was used to calculate the APC rates with their corresponding 95% confidence intervals.

### 2.3. Statistical Formulas Used in the Analysis

- Crude rate: *CIR*_*i*_ = *n*_*i*_/Pop_*i*_∗100.000, where *n*_*i*_ is the number of cancer cases (incidence) in the *i*^th^ age group and Pop_*i*_ is the corresponding local population in person-years.
- ASIR: *ASIR* = ∑CIR_*i*_∗WPop_*i*_/∑WPop_*i*_, where CIR_*i*_ is the crude rate and WPop_*i*_ is the corresponding standard world population for the *i*^th^ age group.
- SE (ASIR): The standard error is calculated with $SE=ASIR/\sqrt{n}$, where *n* is the sample size.
- APC: *APC* = {exp(*b*) − 1}∗100, where *b* is the slope of the regression line estimated by the JoinPoint program.

## 3. Results

**Figure 1:**
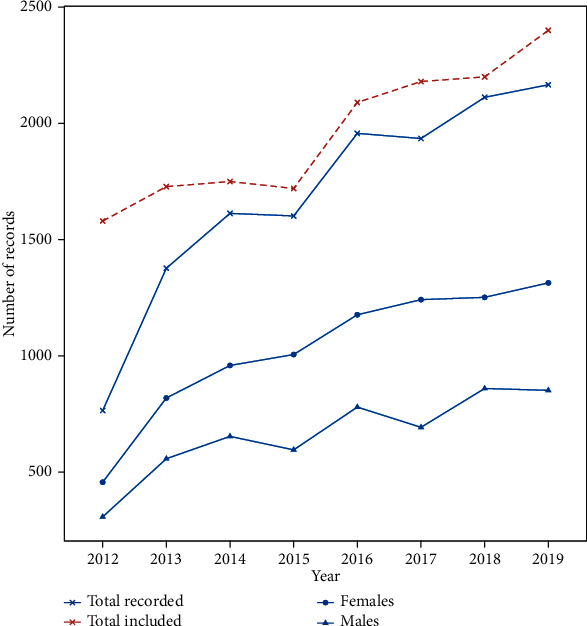
The number of cancer cases admitted and included for registration from 2012 to 2019.

Overall, 15452 new cancer cases were admitted to the Oncological center between 2012 and 2019. Missing data (*n* = 1822) accounted for 11.64%. We excluded patients with brain and CNS cancers (*n* = 353), tumors with an uncertain diagnosis of malignancy (*n* = 6), and duplicated data (*n* = 97). Finally, 13174 cases were selected for statistical analysis and registration (Figure 1).

**Figure 2:**
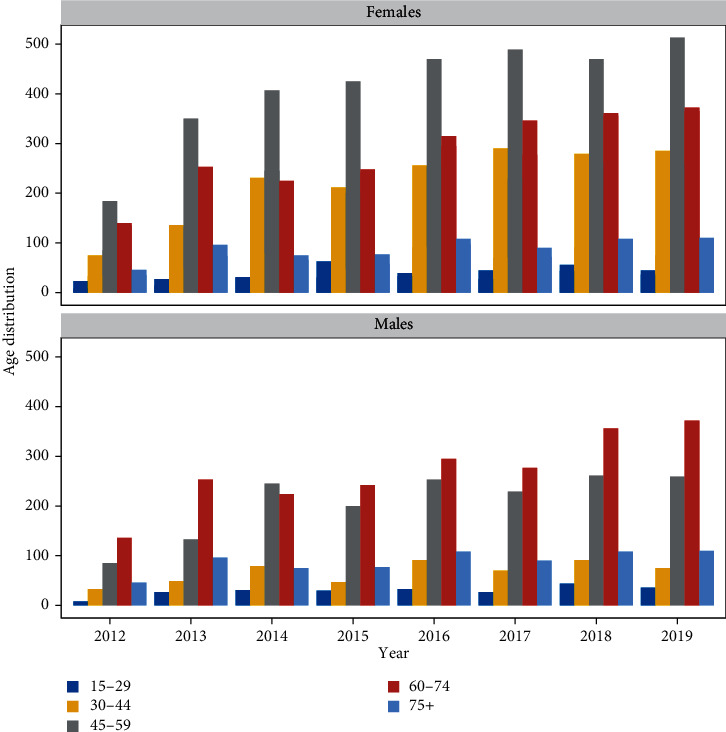
Evolution of cancer cases per age group in both sexes between 2012 and 2019.

The number of cancer cases has been trending upward, with an average annual growth rate of 6.40% throughout the eight years. The median age [interquartile range] at time of diagnosis was 54 [44-63] years for females (mean = 53.98, sd = 13.78) and 61 [52-69] years for males (mean = 59.46, sd = 14.42). The female patients outnumbered their male counterparts (sex ratio = 1.58) among all age groups except those aged ≥75 y. Most cases were reported among the age group 45-59 years (36.69%), followed by the age group 60-74 years (32.69%). The proportion of adults aged ≥75 y and young patients (15-29) accounted, respectively, for 9.85% and 3.81% of all cancer cases and remained stable over the years (Figure 2).

The total annual CIR is 62,00. The female crude rate is 64.31, and the male crude rate is 40.47. The five most common malignancies among females are breast, cervix, ovary, colon, and stomach, followed by endometrial and lung cancers. Among males, the most frequent cancers are as follows: lung, stomach, prostate, colorectal cancers, and both bladder and laryngeal cancers ranked fifth.

The annual ASIR is 68.0 per 100.000 person-years from 2012 to 2019 (SE = 0, 59) and has increased with an APC of 10.61% (95% CI: 4.6-17.0). The ASIR of females is 70,0 (SE = 0, 77) and increases annually with an APC of 10.78% (95% CI: 4.6-17.3). The annual ASIR of males is 45,0 (SE = 0, 63) with an APC of 10.35% (95% CI: 4.0-17.1).

**Figure 3:**
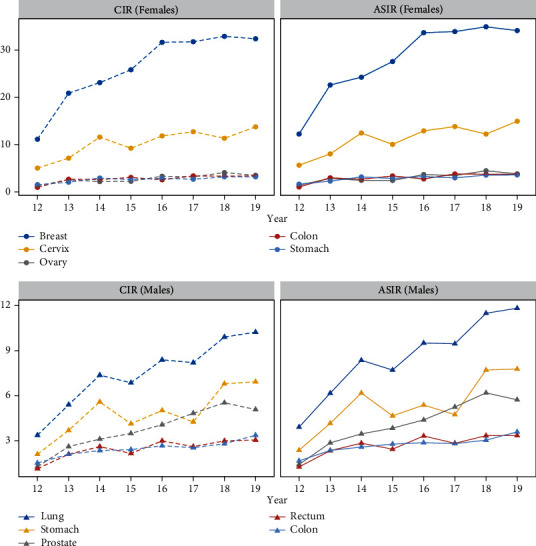
Evolution of annual incidence rates (CIRs and ASIRs) of the five most frequent cancer sites.

The number of cancer cases, percentages, CIRs, and ASIRs in total, in females, and in males for each cancer site are presented in Table 1. The evolution of annual incidence rates (CIRs and ASIRs) of the five most frequent cancer sites is depicted in Figure 3.

The ASIRs of the most common cancers in both sexes, except for colon in females, have increased significantly through the years (Table 2). The ASIR of female breast cancer has shown a significant increase with an APC of +10.85 (95% CI: 3.9-18.2). A pronounced tendency to increase was observed for cervix uteri cancer (APC: +9.85, 95% CI: 2.4-17.8) and ovary cancer (APC: +11.78, 95% CI: 3.8-20.4).

Lung cancer in males has significantly trended up between 2012 and 2019 (APC: +16.2, 95% CI: 8.0-25.0). Stomach cancer has also evolved with an APC = 11.65 (95% CI: 2.5-21.6). Prostate cancer has the maximum significant change with an APC = 16.35 (95% CI: 9.1-24.0), while the minimum significant change was observed in colon cancer with an APC = 7.98 (95% CI: 4.1-12.0). The trend in rectum cancer has also significantly increased with an APC = 8.81 (95% CI: 1.4-16.8) (Table 2).

## 4. Discussion

The estimated ASIR per 100.000 person-years for all sites, including non melanoma skin cancers, is 68.0, which is approximately in line with the rates reported from the two existing PBCRs (Casablanca and Rabat). Females tend to have the highest burden of disease in Marrakech (ratio F/M = 1.58) oppositely to Rabat (ratio F/M = 0.88), while it approaches 1.03 in Casablanca. Remarkably, the median age of cancer diagnosis is 7.0 years lower in females than in males, mainly due, at least in part, to earlier onset of female-related cancers (breast, cervix) compared to lung and prostate cancer among males.

Based on our data, the distribution of cancer incidence follows the same patterns in the three regions (Casablanca, Rabat, and Marrakesh) with similar trends in Europe [10]. The five most common cancers remain the same (breast, colorectal, prostate, and lung cancers) and as in Europe form more than half of the registered cases. These features are different from those observed in Central and Southern African countries, where infection-related cancers are overriding [11]. The overall tendency in Morocco appears to be similar to North African countries, except for Egypt, where liver and bladder are still the most common cancers due to the highest prevalence of hepatitis C and urinary schistosomiasis infection [12, 13].

In North Africa—except for Egypt—breast cancer is the most common malignancy among women, representing 27% to 50% of all female cancers with an estimated annual ASIR ranging between 22.3 in Algeria (2016) [14] and 31.8 in Tunis (2012) [15]. However, such differences are to be interpreted with caution since they take no account of period registration effect and time trends on incidence and might be partly attributable to differences in quality and completeness of registration across borders. For clarity, the most recent reports from Casablanca and Rabat date back to 2012, while our register includes cancer cases from 2012 to 2019 with an overall ASIR that increased by 10.61% over the eight years. The impact of time on incidence trends is *de facto* expected to be considerable.

On another note, the persistence of inequalities and regional disparities in elementary socioeconomic indicators (income, education level, rate of unemployment, poverty and living standards, and access to healthcare) leads to an inequitable distribution of resources with a direct impact on populations of most disadvantaged regions. This fact may yield substantive differences in cancer incidence between the south and non-south populations [16]. Such peculiar features are almost self-explanatory but need further investigation to better assess their effect.

Noteworthy, the incidence of breast cancer is considerably lower compared to developed countries (Europe and USA) and occurs mainly in younger women [17]. These differences can have several interpretations, but they may refer, in particular, to the worldwide marked distribution of breast cancer established risk factors and the possibility of genetic predisposition to breast cancer among native African women. A proof of the validity of our results is the highest proportion of young patients in case series and observational studies reported from North Africa, while it does not exceed 20% in developed countries [18].

Although it is relatively known that reproductive risk factors cannot fully explain the geographic differences, as in North Africa, reproductive behaviors have changed expeditiously in the past decades. In Morocco, lifetime fertility rates are four times lower today than in the past (5.7 in 1980, 2.59 in 2011, and 2.38 in 2018 [Ministry of Health, Morocco National Survey on Population and Family Health]). Other societal changes have been observed such as delayed age at first pregnancy, earlier age at menarche, shorter duration of breastfeeding (17.5 months in 1980 compared to 16.3 months in 2011), growing overweight (29.0% in 1999 to 47.8% in 2000) [19], and the increasing use of oral contraceptives (22.9% in 1987 to 48.4% in 2011and 48.7% in 2018). It has yet to be established whether these continuous changes in procreative health may give insights into the future incidence trends.

Substantial efforts have been devoted to implement mass screening programs of breast cancer in North African countries. Nonetheless, financial constraints to counter the increasingly heavy burden of the disease get more ponderous. A study conducted by the WHO and published in 2003 has provided the first data on screening adherence among young women, revealing that only 2.1% of women above 40 had ever had a screening mammogram in their life [20].

The Breast Cancer Screening Program was established in Morocco in 2010. Women between 40 and 69 years are screened at the primary health centers with a clinical breast examination. A comprehensive evaluation of the program was conducted in 2016–2017 for quality assurance and midterm course correction. It reported that in 2015 and 2016, 1.1 and 1.5 million women were screened, respectively. The program covered 62.8% of the annual target population, clinical examination positivity was 3.2%, and the breast cancer detection rate was 1/1000 women. Marrakesh region was ranked sixth. Overall, the screening coverage was moderate, and the cancer detection rate was low [21].

Despite significant advances in the primary prevention of lung cancer, the incidence of this deadly disease continues to increase in developing countries. A 2017 article from the Regional Oncology Center of Oujda, the only healthcare facility for cancer cases management in Eastern Morocco, has reported the distribution trends of cancer incidence in the northeast region. Between 2005 and 2012, the incidence (CIR) of lung cancer in males has increased (from 5.3 to 8.9 per 100,000) with an APC of 9% [22].

It is well established that the lung cancer incidence reflects cigarette smoking prevalence, with smoking accounting for 80–90% of all lung cancer cases [23, 24]. Therefore, the pronounced rising incidence trend reported in our results may be related to the increasing smoking prevalence [25]. The Maroc Tabagisme-MARTA survey is a national cross-sectional study conducted in 2006 using a random sample of the Moroccan population of 9,195 individuals aged 15 years and above. Among the 5959 participants included in the study, 28.5% of men and 2.8% of women reported they are daily smokers [26]. A single national study conducted in 2014 on a large representative sample of the Casablanca region revealed that tobacco use caused one out of six deaths in Casablanca in 2012, and lung cancer, the leading cause of smoking-attributable mortality, accounted for 34% of it [27].

The rising rates of tobacco use and exposure to work-related chemical carcinogens question the effectiveness of prevention programs and regulatory approaches of occupational exposure as to whether they had a significant positive impact on declining incidence rates. The persistent challenge in all African countries is not related only to the shortage in primary prevention. It also concerns the inaccessibility and the exorbitant cost of recent advances in the management and treatment of lung cancer in the era of personalized and precision medicine.

The Cancer Epidemiology Education in Special Populations (CEESP) has supported a program through funding from the National Cancer Institute in 2015 to conduct a study on the incidence of gastric cancer in Marrakech and Casablanca [28]. Using medical records from Center Hospital University of Marrakech and reports from the cancer register of Casablanca region for the period 2008-2012, Marrakech was found to have a higher age-specific incidence rate of 5.50 compared to 3.23 per 100,000 in Casablanca, which is consistent with our results. Overall, the study revealed interesting observations in the distribution patterns of gastric cancer but could not reliably approach the causal factors behind it. It concluded that further studies are needed to estimate the real burden of cancer in the Marrakech region, explore its risk profile, and provide a comprehensive picture of the possible regional differences.

Mohammed VI University Hospital is the principal healthcare facility for the treatment and management of cancer cases, not only in the region of Marrakech but in all southern regions of Morocco. The main purpose of this study is to report the cancer incidence rates from these unrepresented and most disadvantaged regions. These results are important as they provide significant insights into cancer control and prevention plans in South Morocco. The increasing cancer incidence observed in our results might be a consequence of the change in risk factors or the improved access to formal healthcare and the expanding medical care coverage. However, we are not yet aware of all ongoing changes and how they affect the rising cancer incidence, nor how to adapt our diagnostic capabilities in the upcoming years. Hence, further epidemiological studies are needed to develop evidence-based interventions and address locally effective cancer control plans. In addition, it is fundamental to establish a population cancer registry aggregating data from all public and private facilities in the region to enhance the relevance of output incidence rates.

## 5. Quality of Data and Study Limitations

The underregistration bias and data loss are diminishing the completeness of our data. Cancer patients from the local population may have been diagnosed or managed outside the region or in private healthcare facilities. Likewise, a substantial number of cancer patients may have never been admitted into the formal health system and die without diagnosis or documentation. Therefore, our results may underestimate the real burden of cancer in Marrakech and its surrounding regions.

Moreover, in our referral hospital, pediatric malignancies, tumors of the hematopoietic and lymphoid tissues, and most thyroid cancers are treated, respectively, in pediatric oncology, hematology, and nuclear medicine-specialized departments. Referred patients elected for exclusive radiation therapy, including brain and CNS tumors, are not recorded in the dataset. Thus, in the absence of a shared registry of all departments dealing with cancer diagnosis and treatment, we had only access to the medical records of the patients for whom chemotherapy was indicated.

The manual registration of cancer cases using traditional means (paper) contributes to data loss over time. This trend can be seen in Figure 1, where the missing data in 2012 (*n* = 775) is about 42.53% of the total missing records, while the missing data in the last five years (*n* = 596) was just 32.7%.

The transition from paper-based to computer-based registration will also ease the task of researchers by avoiding the time- and effort-consuming burden of data collection and preprocessing. However, the amount of data included in our study is correlated with the total recorded data (Figure 1), which supports the reliability of our dataset. In addition, the lack of mortality data does not allow approaching the real burden of the disease.

This report highlights the need for further analysis of available data to examine whether the evolution of cancer incidence is driven by a long-term increase in patient volume and what latent variables still need to be ascertained.

## 6. Conclusion

This paper is the first published incidence data of cancer patients from the tertiary care center of Marrakesh. The higher incidence observed in our results reflects a growing burden on the center and is expected to impact our ability to deliver cancer care. The strains on the entire healthcare system will continue to increase if no effective actions are carried out.

Owing to the enormous impact of modifiable risk factors, especially for the most incident cancers, additional efforts are needed to improve cancer prevention programs. Because of its complexity, understanding the cancer distribution across the country requires integrating a mixed combination of lifestyle, health-related risk profiles, and social disparities that might provide interesting insights for future research.

## Figures and Tables

**Table 1:** The number of cancer cases, percentages, crude rates, and ASIRs for each cancer site.

Cancer	Sex														
	All					Females					Males				
	*N*	%	CIR	ASIR	SE	N	%	CIR	ASIR	SE	*N*	%	CIR	ASIR	SE
Breast	3392	25,75	13,49	14,34	0,25	3294	40,77	26,22	27,93	0,49	98	1,92	0,78	0,84	0,08
Cervix	1298	9,85	10,33	11,26	0,31	1298	16,07	10,33	11,26	0,31	—	—	—	—	—
Lung	1105	8,39	4,39	5,05	0,15	165	2,04	1,31	1,49	0,12	940	18,45	7,47	8,55	0,28
Stomach	936	7,10	3,72	4,15	0,14	330	4,08	2,63	2,90	0,16	606	11,89	4,81	5,37	0,22
Colon	655	4,97	2,60	2,86	0,11	344	4,26	2,74	3,00	0,16	311	6,10	2,47	2,71	0,15
Rectum	569	4,32	2,26	2,48	0,10	260	3,22	2,07	2,23	0,14	309	6,06	2,45	2,72	0,15
Prostate	472	3,58	3,75	4,14	0,19	—	—	—	—	—	472	9,26	3,75	4,14	0,19
Ovary	350	2,66	2,79	3,06	0,16	350	4,33	2,79	3,06	0,16	—	—	—	—	—
Bladder/urinary tract	313	2,38	1,24	1,41	0,08	39	0,48	0,31	0,37	0,06	274	5,38	2,18	2,45	0,15
Larynx	307	2,33	1,22	1,38	0,08	33	0,41	0,26	0,28	0,05	274	5,38	2,18	2,46	0,15
Nasopharynx	303	2,30	1,20	1,27	0,07	103	1,27	0,82	0,86	0,08	200	3,93	1,59	1,67	0,12
Nonmelanoma skin	295	2,24	1,17	1,26	0,07	113	1,40	0,90	0,97	0,09	182	3,57	1,45	1,55	0,11
Oesophagus	269	2,04	1,07	1,17	0,07	135	1,67	1,07	1,17	0,10	134	2,63	1,06	1,17	0,10
Endometrium	238	1,81	0,95	1,08	0,07	228	2,82	1,81	2,09	0,14	10	0,20	0,08	0,09	0,03
Pancreas	234	1,78	0,93	1,06	0,07	109	1,35	0,87	0,99	0,09	125	2,45	0,99	1,13	0,10
Unclear	199	1,51	0,79	0,85	0,06	106	1,31	0,84	0,89	0,09	93	1,83	0,74	0,81	0,08
Bone cartilage	163	1,24	0,65	0,67	0,05	66	0,82	0,53	0,52	0,06	97	1,90	0,77	0,82	0,08
Gallbladder	157	1,19	0,62	0,72	0,06	116	1,44	0,92	1,08	0,10	41	0,80	0,33	0,36	0,06
Soft tissue	137	1,04	0,54	0,56	0,05	74	0,92	0,59	0,60	0,07	63	1,24	0,50	0,52	0,06
Thyroid gland	135	1,02	0,54	0,59	0,05	88	1,09	0,70	0,76	0,08	47	0,92	0,37	0,41	0,06
Cancer of unknown primary CUP	122	0,93	0,49	0,53	0,05	58	0,72	0,46	0,51	0,07	64	1,26	0,51	0,56	0,07
Vulva	112	0,85	0,89	0,98	0,09	112	1,39	0,89	0,98	0,09	—	—	—	—	—
Kidney	110	0,83	0,44	0,49	0,05	35	0,43	0,28	0,31	0,05	75	1,47	0,60	0,67	0,08
Tongue	109	0,83	0,43	0,46	0,04	54	0,67	0,43	0,46	0,06	55	1,08	0,44	0,46	0,06
Liver	95	0,72	0,38	0,42	0,04	40	0,50	0,32	0,36	0,06	55	1,08	0,44	0,48	0,06
Anus	85	0,65	0,34	0,37	0,04	25	0,31	0,20	0,21	0,04	60	1,18	0,48	0,52	0,07
Placenta	72	0,55	0,57	0,57	0,07	72	0,89	0,57	0,57	0,07	—	—	—	—	—
Hodgkin disease	60	0,46	0,24	0,24	0,03	29	0,36	0,23	0,23	0,04	31	0,61	0,25	0,25	0,04
Melanoma of skin	55	0,42	0,22	0,24	0,03	25	0,31	0,20	0,22	0,04	30	0,59	0,24	0,25	0,05
Nasal/paranasal sinuses	50	0,38	0,20	0,22	0,03	18	0,22	0,14	0,16	0,04	32	0,63	0,25	0,28	0,05
Hypopharynx	47	0,36	0,19	0,20	0,03	23	0,28	0,18	0,20	0,04	24	0,47	0,19	0,21	0,04
Parotid	47	0,36	0,19	0,21	0,03	18	0,22	0,14	0,15	0,03	29	0,57	0,23	0,26	0,05
Testis	47	0,36	0,37	0,38	0,05	—	—	—	—	—	47	0,92	0,37	0,38	0,05
Lip	41	0,31	0,16	0,18	0,03	13	0,16	0,10	0,11	0,03	28	0,55	0,22	0,24	0,05
Biliary tract	40	0,30	0,16	0,18	0,03	23	0,28	0,18	0,20	0,04	17	0,33	0,14	0,15	0,04
Small intestine	40	0,30	0,16	0,18	0,03	22	0,27	0,18	0,20	0,04	18	0,35	0,14	0,15	0,04
Appendix	39	0,30	0,16	0,16	0,03	22	0,27	0,18	0,19	0,04	17	0,33	0,14	0,14	0,03
Mouth	39	0,30	0,16	0,17	0,03	24	0,30	0,19	0,22	0,04	15	0,29	0,12	0,13	0,03
Uteri	38	0,29	0,30	0,31	0,05	38	0,47	0,30	0,31	0,05	—	—	—	—	—
Ear	29	0,22	0,12	0,11	0,02	8	0,10	0,06	0,06	0,02	21	0,41	0,17	0,16	0,04
Peritoneum	28	0,21	0,11	0,13	0,02	16	0,20	0,13	0,15	0,04	12	0,24	0,10	0,11	0,03
Eye and adnexa	27	0,20	0,11	0,11	0,02	15	0,19	0,12	0,13	0,03	12	0,24	0,10	0,10	0,03
Oropharynx	27	0,20	0,11	0,12	0,02	7	0,09	0,06	0,06	0,02	20	0,39	0,16	0,18	0,04
Thymus	23	0,17	0,09	0,10	0,02	10	0,12	0,08	0,09	0,03	13	0,26	0,10	0,11	0,03
Vagina	22	0,17	0,18	0,18	0,04	22	0,27	0,18	0,18	0,04	—	—	—	—	—
NHL	21	0,16	0,08	0,09	0,02	6	0,07	0,05	0,05	0,02	15	0,29	0,12	0,13	0,03
Tonsil	20	0,15	0,08	0,09	0,02	9	0,11	0,07	0,08	0,03	11	0,22	0,09	0,10	0,03
Pleura	17	0,13	0,07	0,08	0,02	6	0,07	0,05	0,05	0,02	11	0,22	0,09	0,10	0,03
Adrenal gland	15	0,11	0,06	0,07	0,02	8	0,10	0,06	0,07	0,03	7	0,14	0,06	0,06	0,02
Neural crest tumors	15	0,11	0,06	0,06	0,01	4	0,05	0,03	0,03	0,01	11	0,22	0,09	0,09	0,03
Plasmocytoma	15	0,11	0,06	0,07	0,02	7	0,09	0,06	0,06	0,02	8	0,16	0,06	0,07	0,03
Salivary glands	15	0,11	0,06	0,07	0,02	10	0,12	0,08	0,09	0,03	5	0,10	0,04	0,04	0,02
Sarcoma-unspecified	14	0,11	0,06	0,06	0,02	5	0,06	0,04	0,04	0,02	9	0,18	0,07	0,08	0,03
Unspecified lymphoma	13	0,10	0,05	0,05	0,01	7	0,09	0,06	0,06	0,02	6	0,12	0,05	0,05	0,02
Meningeal	10	0,08	0,04	0,04	0,01	7	0,09	0,06	0,06	0,02	3	0,06	0,02	0,02	0,01
Multiple myeloma	10	0,08	0,04	0,04	0,01	6	0,07	0,05	0,05	0,02	4	0,08	0,03	0,03	0,02
Duodenum	9	0,07	0,04	0,04	0,01	5	0,06	0,04	0,04	0,02	4	0,08	0,03	0,03	0,02
Peripheral nerves	9	0,07	0,04	0,03	0,01	4	0,05	0,03	0,03	0,02	5	0,10	0,04	0,04	0,02
GIST-unspecified	8	0,06	0,03	0,04	0,01	5	0,06	0,04	0,05	0,02	3	0,06	0,02	0,03	0,01
Kaposi	8	0,06	0,03	0,03	0,01	1	0,01	0,01	0,01	0,01	7	0,14	0,06	0,06	0,02
Connective tissue	7	0,05	0,03	0,03	0,01	1	0,01	0,01	0,01	0,01	6	0,12	0,05	0,05	0,02
Ganglia-unspecified	7	0,05	0,03	0,03	0,01	1	0,01	0,01	0,01	0,01	6	0,12	0,05	0,05	0,02
Mesentery	7	0,05	0,03	0,03	0,01	1	0,01	0,01	0,01	0,01	6	0,12	0,05	0,05	0,02
Paraganglias	7	0,05	0,03	0,03	0,01	3	0,04	0,02	0,03	0,02	4	0,08	0,03	0,03	0,01
Penis	7	0,05	0,06	0,07	0,03	—	—	—	—	—	7	0,14	0,06	0,07	0,03
Mediastinum	5	0,04	0,02	0,02	0,01	1	0,01	0,01	0,01	0,01	4	0,08	0,03	0,03	0,02
Parathyroid gland	2	0,02	0,01	0,01	0,01	2	0,02	0,02	0,02	0,01	0	0,00	0,00	0,00	—
Urachus	2	0,02	0,01	0,01	0,01	0	0,00	0,00	0,00	—	2	0,04	0,02	0,02	0,01
Total	13174	100	62,00	68	0,59	8079	100	64,31	70	0,77	5095	100	40,47	45	0,63

**Table 2:** Trends in ASIRs of the five most frequent cancers in both sexes. The asterisk indicates that the APC is significantly different from 0.

Cancer	Sex	APC
Breast	F	10.85 [3.9; 18.2]^∗^
Cervix	F	9.85 [2.4; 17.8]^∗^
Lung	M	16.2 [8.0; 25.0]^∗^
Stomach	F	11.2 [3.5; 19.5]^∗^
	M	11.65 [2.5; 21.6]^∗^
Colon	F	9.26 [-0.4; 19.9]
	M	7.98 [4.1; 12.0]^∗^
Rectum	M	8.81 [1.4; 16.8]^∗^
Prostate	M	16.35 [9.1; 24.0]^∗^
Ovary	F	11.78 [3.8; 20.4]^∗^
All sites	F	10.78 [4.6; 17.3]^∗^
	M	10.35 [4.0; 17.1]^∗^
	Both sexes	10.61 [4.6; 17.0]^∗^

## Data Availability

N. Benhima and A. Laatabi have full access to data and are responsible for its integrity and accuracy. Data can be made available upon reasonable request with the permission of authors.
